# Analysis of Thermally Induced Residual Stress in Resistance Welded PC/CF Composite to Aluminum

**DOI:** 10.3390/ma18214962

**Published:** 2025-10-30

**Authors:** Marcin Praski, Piotr Kowalczyk, Karolina Stankiewicz, Radosław Szumowski, Piotr Synaszko, Andrzej Leski

**Affiliations:** 1 Faculty of Mechanical Engineering, Military University of Technology, 01-480 Warsaw, Poland; andrzej.leski@ilot.lukasiewicz.gov.pl; 2 Lukasiewicz Research Network–Institute of Aviation, 02-256 Warsaw, Poland; piotr.kowalczyk@ilot.lukasiewicz.gov.pl (P.K.); karolina.stankiewicz@ilot.lukasiewicz.gov.pl (K.S.); radoslaw.szumowski@ilot.lukasiewicz.gov.pl (R.S.); 3 Air Force Institute of Technology, 01-494 Warsaw, Poland; piotr.synaszko@itwl.pl

**Keywords:** thermography, fem, thermoplastic composite, polycarbonate, residual stress, dissimilar material joints

## Abstract

Thermoplastic composites are growing in popularity in the aerospace and automotive industries; they enable weldable and recyclable structures. Resistance welded hybrid thermoplastic and metal joints are attractive for rapid assembly, but the thermal mismatch between metals and polymers introduces residual stresses, which can drive edge debonding and compromise durability. This study presents fabricated single-lap PC/CF–Al7075 coupons with measured mid-span bow resulting from welding, evaluated bond quality by step-heating thermography, and an evaluated framework for residual stress prediction using Ansys complemented by a bimetal analytical check. Three thermal cycles were examined with different temperature gradients (200, 220, 240 °C): the measured bow was 16.5 mm and remained constant, whereas analytical calculation increased with ΔT similarly to the FEM prediction. The current FEM under predicted the bow (Mean Absolute Percentage Error is 21%), showing stress contours that decay with distance from the bond and revealing pronounced peaks in both σ_xx_ and σ_zz_ components at weld edges, consistent with shear-lag theory. FEM returned edge-peaked peel rising from 43 to −64 MPa and σ_xx_ was up to 12% more compressive than analytical calculation; an effective CF/PC CTE of 1.5 × 10^−6^ K^−1^ reconciled curvature with test better than catalogue values. The temperature insensitive bow is attributed to polycarbonate flow/viscoelastic relaxation above Tg and hot relaxation in aluminum, with effects not represented in the elastic models. Edge peel and shear govern initiation risk.

## 1. Introduction

In recent years, thermoplastic composites, most importantly the carbon fibre-reinforced systems, have grown significantly in popularity in industries such as aerospace and automotive because they combine the benefits that traditional carbon fibre composites carry and improve on them by adding short manufacturing cycles, weldability, repairability, and recyclability [1], while offering similar structural performance as well as increased damping and the potential for decreased manufacturing energy requirements, this novel thermoplastic material is a promising replacement for thermoset epoxy materials, leading to a reduction in overall production costs in industries focused on efficiency and sustainability [2,3,4,5]. Amorphous polymers such as polycarbonate exhibit strong temperature-dependent stiffness around the glass transition, which enables thermal joining but also makes the thermal cycle a driver of post process deformations and residual stress [6]. Effective application requires advanced joining techniques that will ensure structural integrity and confidence in fatigue resistance. The combination of a polymer matrix and reinforcing fibres results in high-performance materials [7].

Recognizing the benefits and accelerating adoption of thermoplastic composites, this study addresses a research question: can carbon fibre-reinforced polycarbonate (PC/CF) be reliably welded to aluminum to form strong bonds and can the process be repeatable enough for structural service and repair? Prior to testing the joint strength and durability, this paper focuses on the risk of the dominant manufacturing side effect, the residual stresses arising from the weld thermal cycle, and subsequent cooling, driven by coefficient of thermal expansion (CTE) mismatch and temperature-dependent stiffness.

Recent studies on CFRP to metal welds quantified residual stress magnitudes, while simulations reproduced the thermos mechanical fields that drive interface failure. Collectively, these studies motivate a validated thermal structural workflow for predicting post-weld deformation and interfacial stress in thermoplastic metal joints [8,9]. We create a representative PC/CF to aluminum singe-lap joint and execute a validated two stage finite element workflow to anticipate and quantify post-weld deformation and the magnitude, spatial distribution, and edge-concentrated peel and shear that signal incipient debonding. The objective here is not to estimate the joint strength but to provide evidence on the feasibility of welding thermoplastic to metal and on the process parameters that govern residual stresses like temperature profile, pressure, and time, as shown by Sacchetti et al., controlling the cooling rate can significantly improve composite fatigue strength by up to 2.5 times [10]. establishing a technical baseline for subsequent strength, durability, and repair use cases.

Prior work on joining thermoplastic composites to metals, resistance, and induction variants has established process parameters and post-weld assessments [11,12,13,14], yet validated residual stress predictions in thermoplastic–metal single-lap welds remain sparse, particularly for PC/CF to Al [15]. Existing studies emphasize process feasibility and joint morphology [16]; fewer provide a quantitative link between measured thermal histories, temperature-dependent property cards, and post-cooling residual stresses benchmarked against deformation and NDT [8,17]. This motivates the present focus on residual stress quantification and validation ahead of strength and durability. Similar durability study is well documented in metallic stir friction welds [18]. It reports that accelerated crack propagation under tensile residual stress, including residual stress in an analysis, changes the expected strength of the joint, which can be seen in [19].

There is limited data on validated residual stress prediction for carbon fibre polycarbonate bonded with aluminum. Much of the literature emphasizes process feasibility and joint morphology, whereas fewer studies quantitatively link measured or modelled thermal histories, temperature-dependent property cards, and post-cooling residual stresses benchmarked against deformation or NDT. Accordingly, we model the weld thermal field with temperature-dependent material cards [20,21,22] and propagate that history to a structural analysis to estimate deformation and residual stresses, then compare predictions with the deformed specimen and weld-quality measurements (thermography); the thermal results are implemented to a structural analysis to estimate the deformation and resulting stress. This is compared with the actual welded and deformed specimen. Polycarbonate was chosen due to its relatively low transition temperature which should minimize the post-weld residual stress. Polycarbonate is selected for its relatively low glass transition temperature, which can reduce post-weld residual stress risk for a given thermal input.

**Figure 1:**
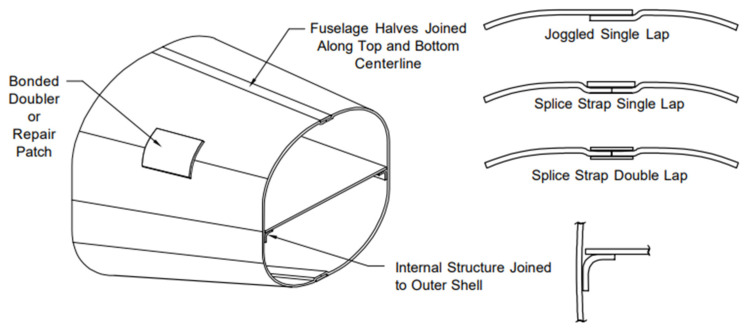
Typical examples of SLJ usage [24].

Two plates were joined using a single-lap joint (Figure 1). The simplicity of this joint makes it versatile and widely used in the aerospace industry (Figure 1) [23].

## 2. The Experiment

### 2.1. Materials and Specimens

Two panels were joined in a single-lap configuration, a carbon fibre-reinforced polycarbonate laminate (PC/CF) and an aluminum alloy (7075) plate. A 0.2 mm polycarbonate film was used as a welding interlayer. Nominal dimensions of the specimens are summarized in Table 1; material property data and temperature dependencies used later in the FE model are described in Section 5.

### 2.2. Surface Preparation

**Figure 2:**
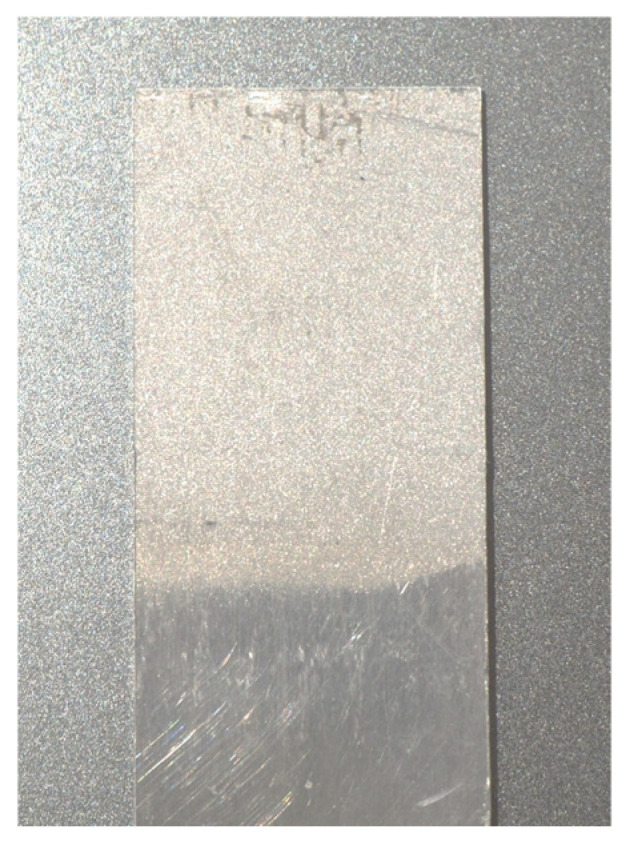
Aluminum plate surface preparation.

**Figure 3:**
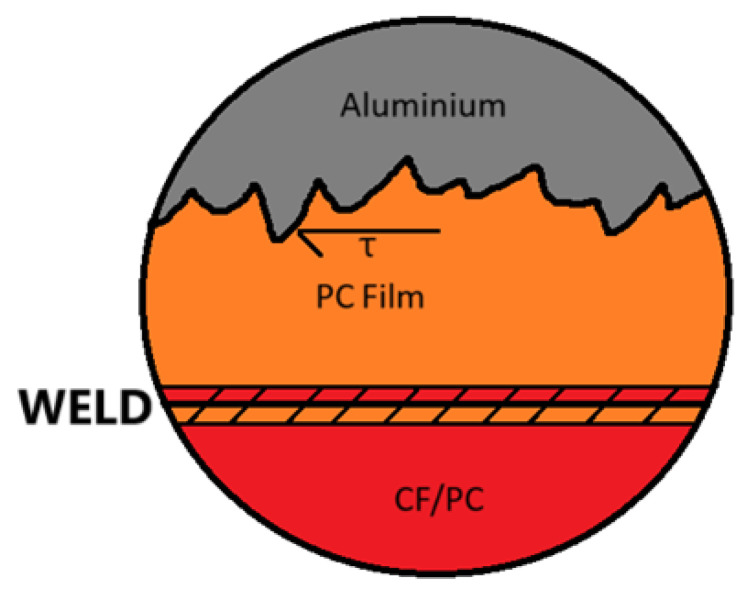
The influence of the surface roughness on the tangential stress.

To ensure better joint strength, the surface of the aluminum plate is prepared beforehand (Figure 2). Baker A.A. describes that when controlled micro roughness and a chemically active oxide are used to enhance wetting, promote micromechanical interlocking, and improve durability, the mechanical interlocking of the aluminum with molten polycarbonate introduces an additional shear force component (Figure 3) that increases the shear strength of the joint [25]. Furthermore, the intricate structure of the surface improves durability and moisture resistance, as the complex shape of the joint prolongs the path for water penetration. However, over blasting the aluminum surface traps contaminants and moisture and degrades durability [25]. A corroboration of the surface roughness and adhesion strength relationship is presented in [12], where it is reported that etching of stainless-steel increased surface roughness and raised lap shear strength by up to 32%, with excessive etch time reducing strength due to wire degradation. The review further notes that anodizing aluminum forms a microporous, micro rough oxide that promotes mechanical interlocking with amorphous thermoplastics, improving adhesion relative to smooth metal surfaces. Within [26], a great comparison of surface roughness to adhesion strength is presented, and a value of R_a_ = 10.18 [µm] can be observed. On the composite side, the bond relies on the thermoplastic nature of polycarbonate. This process, often described as welding, involves heating the polycarbonate to its flow temperature. At this stage, inter-chain bonds become weak, and the polymer flows freely [27], allowing the polycarbonate within the composite and the film to mix thoroughly and penetrate the roughness of the aluminum. As the temperature cools below the glass transition point, the thermoplastic hardens, forming a durable bond. For this experiment the aluminum plate surface was prepared using grit blasting following the guidance presented in [25], and the roughness was judged visually and by feel.

### 2.3. Resistance Welding Carbon Fibre Reinforced Polycarbonate with Aluminum

#### 2.3.1. Introduction to Resistance Welding of Thermoplastic Composites

**Figure 4:**
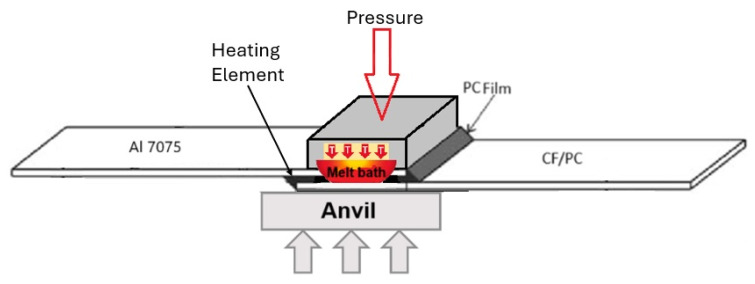
Illustration of the welding process.

Resistance welding generates localized heat through electrical resistance to melt the material at the interface of a joint. This process follows the principles of Joule heating Q = I^2^Rt, as current passes through a resistive element placed between the adherends. This heating element can be a metallic mesh, carbon fibres, or a nanocomposite, each chosen based on its specific resistive properties [12]. A metal mesh tends to deliver uniform heating and a wider processing window and was chosen for this experiment. The joint comprises two components, a carbon fibre-reinforced polycarbonate (PC/CF) and aerospace grade aluminum (AL7075). A polycarbonate film is placed between these two components to facilitate the weld. The goal is to generate enough heat to melt the bonding layers and hold this state long enough to allow the PC film to integrate with the PC matrix and penetrate the aluminum surface roughness. Any excess material should be extruded. But the time should be short enough not to heat the whole laminate, and only the bonding layers, otherwise the composite internal structure will degrade. Reducing time by raising the power will result in burning the top layers and oxidizing the fibres [28,29]. For a proper diffusion, pressure application is necessary [30] (Figure 4). The pneumatic actuators apply three bars pressure to the components being joined; maintaining constant pressure is very important and might be challenging due to the thermal expansion of the heated materials, and thus, a feedback-controlled system is recommended. For the best results, the pressure value must eliminate the elastic deformation of the fibre and cause the excess of the polymer film to leak outside the bond. Excessive pressure will lead to a complete film extrusion and additional partial matrix leakage, ending in a dry bond [28,30]. The right combination of thermal energy, time, and pressure ensures quick and effective welding, with the applied pressure allowing for the extrusion of excess molten film and better bonding [12,28].

**Figure 5:**
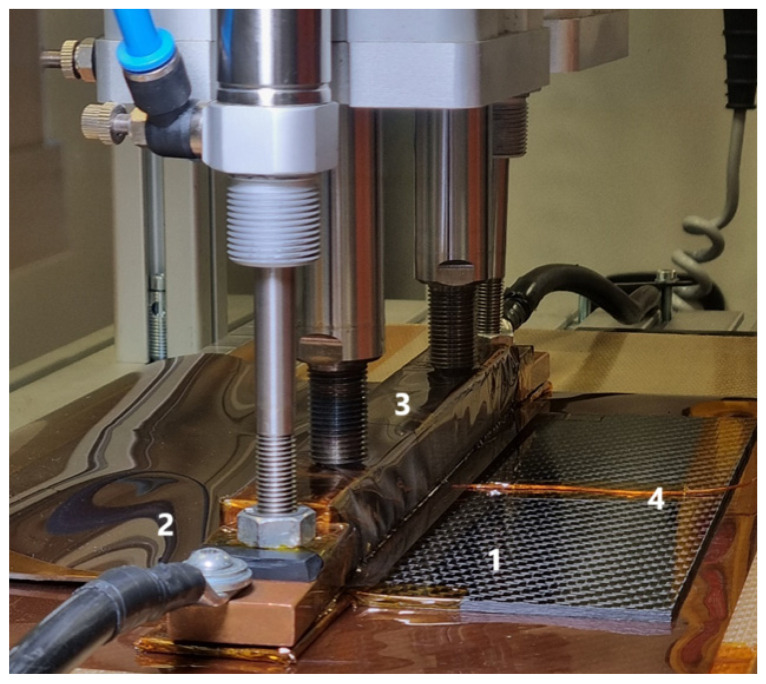
Welding station. 1. PC/CF composite plate, 2. aluminum plate, 3. pneumatic actuator, 4. thermocouple.

The workstation (Figure 5) is composed of the following:

- A worktable serving as an anvil where the bonding process is performed.
- Copper electrical connectors to supply power to the welding machine.
- An aluminum clamp, onto which the press applies force.
- A steel mesh to ensure uniform heating.
- Glass fabric coated with polyimide film, serving as an insulator.

**Figure 6:**
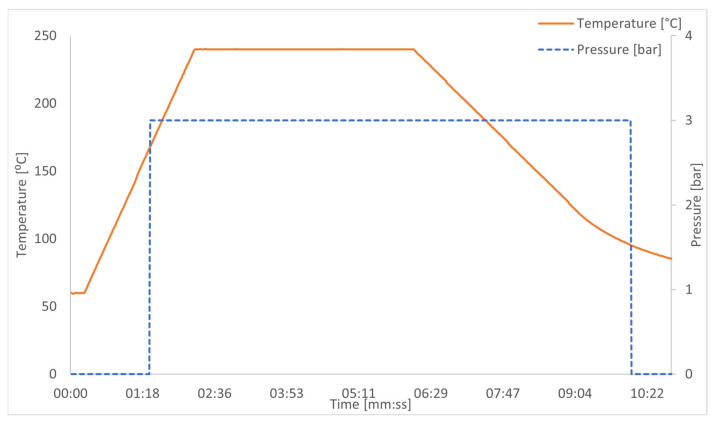
Welding process: temperature [°C], pressure [bar], time [s].

For welding PC composites with aluminum, precise control of parameters such as temperature, heating time, and pressure is crucial. The process consists of several steps:

- Surface Preparation: Aluminum requires proper preparation to ensure good adhesion with molten polycarbonate. This includes mechanical grinding and cleaning of the surface to remove oxides and contaminants [7].
- Setting Welding Parameters: For PC-aluminum joints, it is recommended to set the temperature in the range of 220–260 °C. Exceeding this temperature may lead to polycarbonate degradation, reducing joint strength. Optimal heating time is usually 120–200 s, depending on material thickness and applied pressure, which should be approximately 0.2–0.5 MPa (Figure 6) [7].
- Welding: During heating, electric current flows through the resistive heating element, quickly heating the surfaces of polycarbonate and aluminum. The high temperature causes the polycarbonate to melt, bonding with the aluminum surface and forming a durable connection [7].

#### 2.3.2. The Welding Process

**Figure 7:**
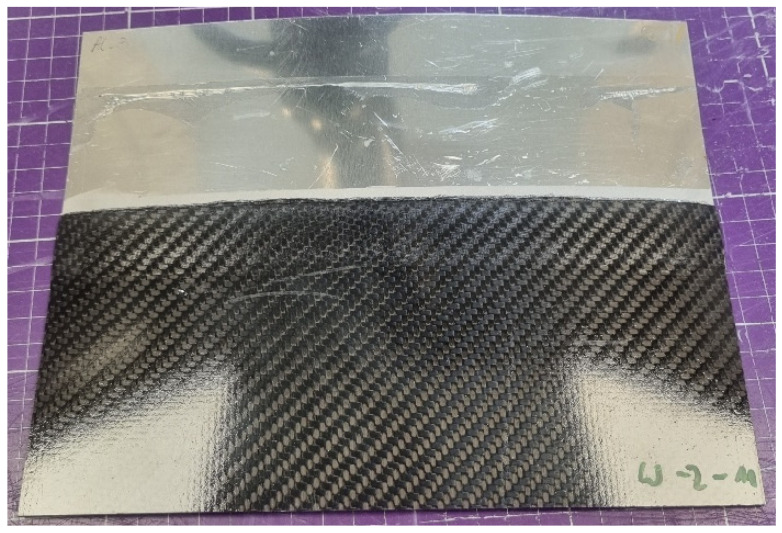
Welded plates.

During the welding process, temperature and pressure control are crucial. Examples of optimal parameters for welding PC with aluminum include the following (Figure 7):

- Welding Temperature: 220–260 °C
- Heating Time: 120–200 s
- Pressure: 0.2–0.5 MPa

#### 2.3.3. Welded Parts

**Figure 8:**
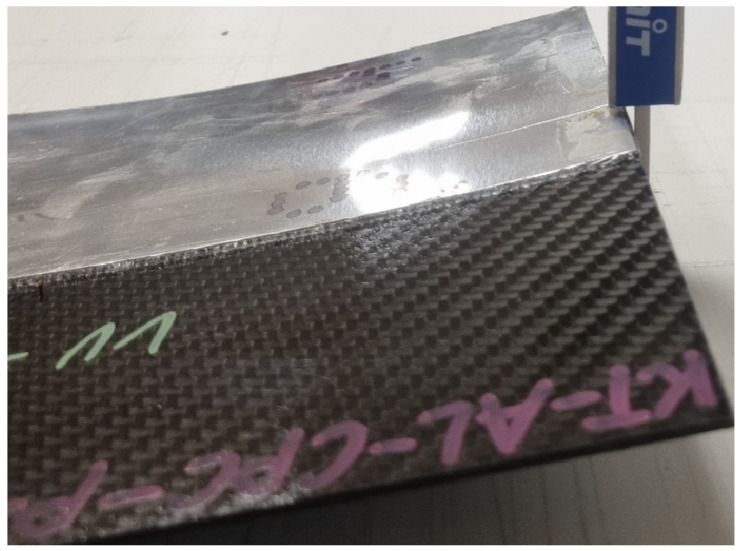
Deformation of the plates due to the residual stress.

In the Lukasiewicz Research Network, Institute of Aviation, plates are joined following internally established processes. The temperature in the initial phase is raised to the flow temperature, at which point the welding process occurs. After the appropriate time, the energy supply is cut off, and the entire workstation is left to cool. Upon cooling, the effect of residual stress becomes apparent (Figure 7 and Figure 8).

Four sample joints were created. Their joint thicknesses, which varied due to the amount of extruded material, was measured. Mid-span sagitta δ over a 240 mm chord at room temperature was measured with a digital calliper (0.01 mm resolution, ±0.02 mm accuracy). Samples were supported on equal-height parallels with a straightedge defining the chord; 10 repeated measurements were taken per specimen without repositioning. The uncertainty remained within U = 0.03–0.07 mm less than 0.5% of δ and far below the specimen scatter. These values will assist in determining the stress that caused it. The joint was subjected to nondestructive testing, and the entire assembly will be cut into smaller samples for subsequent static tensile testing. Across all samples, the mean deformation ranged from 16.5 to 17.0 mm, with low scatter. The maximum coefficient of variation was 3.4%, indicating acceptable repeatability for welded joint deformation.

## 3. Nondestructive Testing Using Thermography

Thermography is one of the most versatile nondestructive testing methods, and the active infrared thermography was used to test the joint region for any voids or incomplete bond. Step heating was chosen because it balances depth reach and simplicity. A comparison of pulse and lock-in thermography is presented in [31,32].

### 3.1. Thermography Testing Using the C-CheckIR

#### 3.1.1. The Description of the Equipment

Thermography testing was performed using the C-CheckIR mobile (AT—Automation Technology GmbH, Bad Oldesloe, Germany) inspection system developed by ECTS. This system is equipped with a thermal imaging camera and heat sources, enabling testing both in laboratory and field conditions. The setup can detect defects in composite materials, such as delamination and voids, without contacting the tested object.

#### 3.1.2. Testing Procedure

Step heating was applied with 1.5 s excitation and 20 s observation per scan. The field of view covered the entire overlap with a 10 mm margin. Stand-off and focus were held constant for all scans. Each specimen was scanned three times without repositioning to quantify repeatability. A bare aluminum patch and the black tape reference were captured in each sequence to check emissivity and reflections. All scans were performed with the CFRP side up.

### 3.2. Sample Preparation

**Figure 9:**
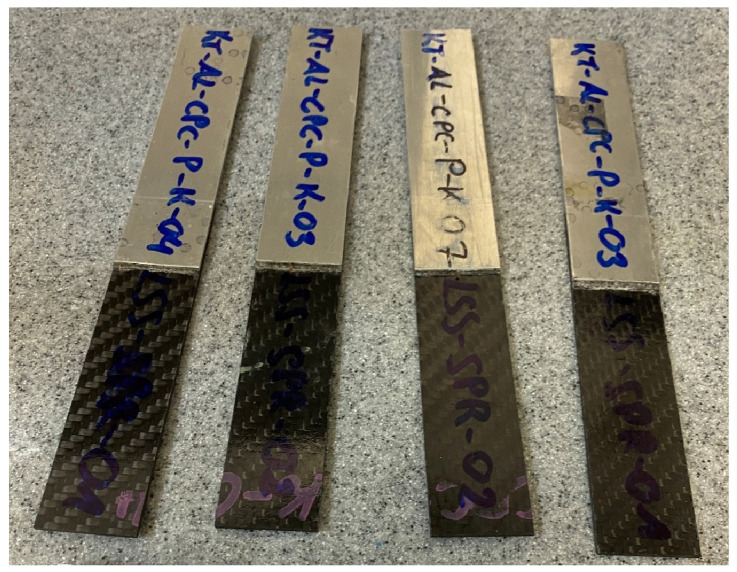
Thermographic testing samples.

Welded plates were cut into smaller specimens and prepared for testing (Figure 9). Each sample was cleaned to minimize the impact of contaminants on the results.

### 3.3. Results

For each specimen, three scans were made, a weld scan was accepted when no voids lager than 1 mm were detected, normalized contrast (C_norm_) remained under 6 in the overlap region, and no visible discontinuities at the edge area were observed.

**Figure 10:**
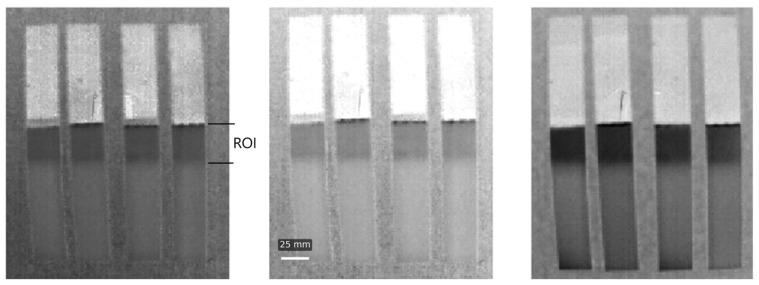
Thermographic pictures of the samples.

Thermographic testing is a great method for finding any voids or other material discontinuities inside specimens. Like any other method this one also comes with its own limitations, namely, the camera resolutions. These are usually too small to detect micropores, which might appear in large numbers, but each individual has a fraction of a millimetre in diameter. If such defects exist, they reduce the effective cross-sectional area of the bond and influence the structural performance of the joint [33]. High precision NDT methods were not within the scope of this work. The joint quality assessment is presented by the method described above and illustrated in (Figure 10).

In the figures above the alloy plate, carbon fibre plate and the lap joint in the middle with its darkest colour can be distinguished. What we are looking for during the thermograph test are any lighter spots within the joint, these would present any discontinuities or air bubbles trapped inside. Such voids raise the driving factors behind the crack initiation, and after initiations helps the propagation by connecting the path between neighbouring pores [34]. A homogeneous dark tone of the lap indicates that full contact has been achieved and both plates are welded correctly.

## 4. Analytical Considerations of the Residual Stress in a Joint

Before conducting finite element simulations, it is good practice to perform simplified hand calculations. This practice should deliver rough estimates of what should appear in the numerical analysis.

### 4.1. Normal Stress

For a welded single-lap PC/CF–Al overlap, the cooled part behaves as a bi-material strip. When this strip is uniformly heated along its entire length, it will bend or deform into an arc of a circle with a radius of curvature, the value of which, is dependent on the geometry and material components making up the strip. This behaviour is the result of a thermal expansion coefficient mismatch. And the subsequent deflection curvature can be estimated using classic Timoshenko, here a Roark’s Formula for Stress and Strain which tabulates engineering forms for thermal curvature and thermal-flexure stress [35] was considered. With these formulas and using the input data presented in Table 2 we can predict the sign and magnitude of curvature κ, the mid-span bow δ, and the membrane stress.

The curvature can be calculated using

(1) $$\kappa =\frac{6\left(\alpha_{b}-\alpha_{a}\right)∆T\left(t_{a}+t_{b}\right)}{t_{b}^{2}\left[4+6r+4r^{2}+\left(\frac{E_{a}}{E_{b}}\right)r^{3}+\left(\frac{E_{b}}{E_{a}}\right)r^{-1}\right]},r=\frac{t_{a}}{t_{b}}$$

(2) $$\delta =\frac{\left|\kappa \right|L^{2}}{8}$$

Shear stress is expressed by

(3) $$\sigma_{xx,i}(z)=E_{i}(\varepsilon_{0}+\kappa z-\alpha_{i}∆T)$$

### 4.2. Results and Conclusions

**Figure 11:**
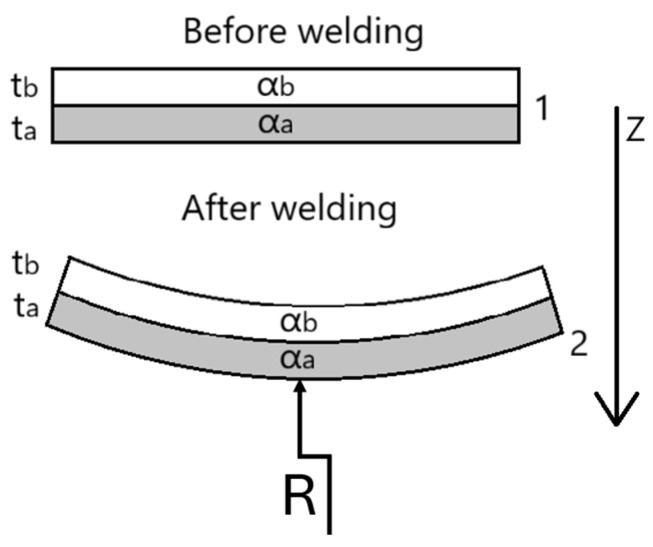
Bi-material strip, pre- and post-welding.

When Z axis begins at the bond interface and is defined towards the PC/CF laminate (Figure 11), the curvatures negative value informs us that the aluminum layer shrinks more upon cooling, bending the strip towards the aluminum face. The negative $\sigma_{xx}$ indicates that the system is in a compressive state after cooling. Full results are presented in Table 3.

The bow δ value for the ΔT = 240 °C is very close to the measured values of the welds; however, these measured values remained unaffected by temperature (Table 3).

Why do the calculated and measured deflection not match? Resistance welding concentrates heat very locally at the bonding interface, under the addition of three bars of pressure. The effective temperature gradient remains mostly in the welded area, meaning the cooling dependent deformation is strongest at the joint area [30,38]. Around the T_g_, polycarbonate experiences full stress relaxation and recovery, which means the thermal mismatch above this point does not contribute to the deformation [39].

## 5. Numerical Model

**Figure 12:**
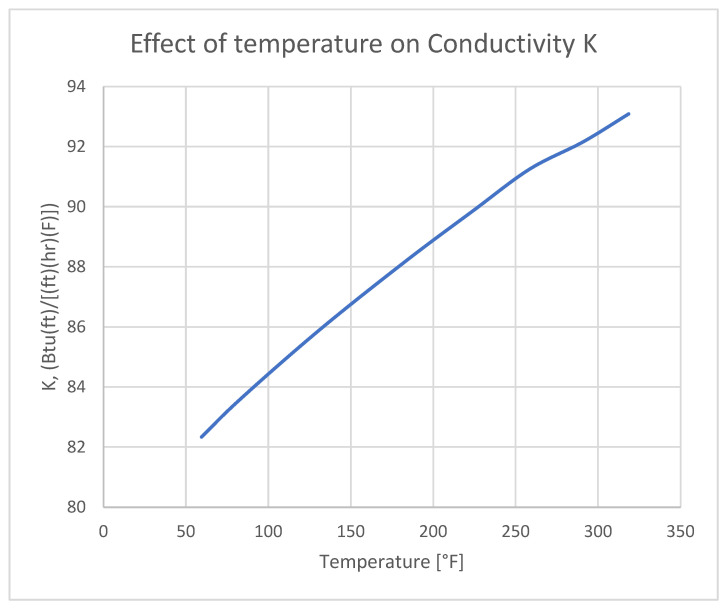
The effect of temperature on the conductivity of aluminum 7075 [41].

**Figure 13:**
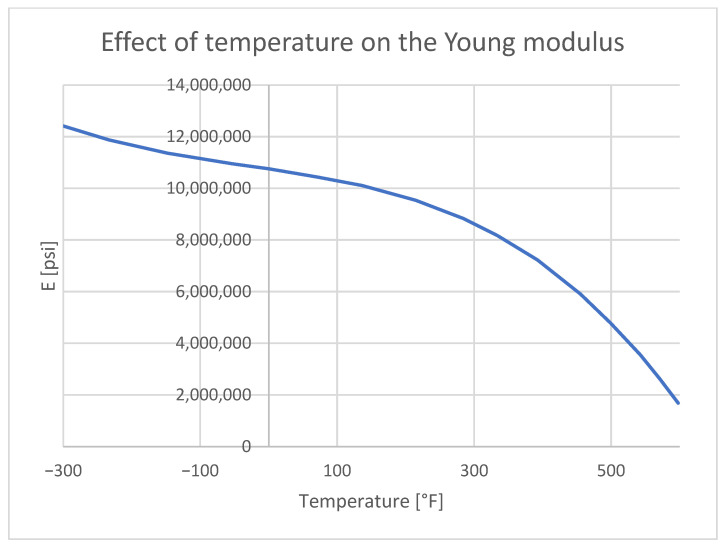
The effect of temperature on the Young modulus on aluminum 7075 [41].

To obtain reliable stress results in thermal analysis, it is essential to introduce detailed material property cards. Materials with non-linear characteristics, with temperature-dependent values, are crucial for achieving accurate results. Below are the characteristics of the input thermal conductivity (Figure 12) and Young’s modulus (Figure 13).

The following graph illustrates how effectively heat moves through a given material when there is a temperature difference between its two sides. The higher the thermal conductivity, the better the material’s ability to transfer heat. This relationship is described by the Fourier’s Equation (4) [40].

(4) $$q=-\frac{\lambda dT}{dx}$$

Specific Heat C (Equation (5)) describes the amount of heat required to raise the temperature of a unit mass of a given material by 1 degree Celsius (or 1 Kelvin). It is a measure of a substance’s capacity to store thermal energy.

(5) $$C=\frac{Q}{m∆T}$$

**Figure 14:**
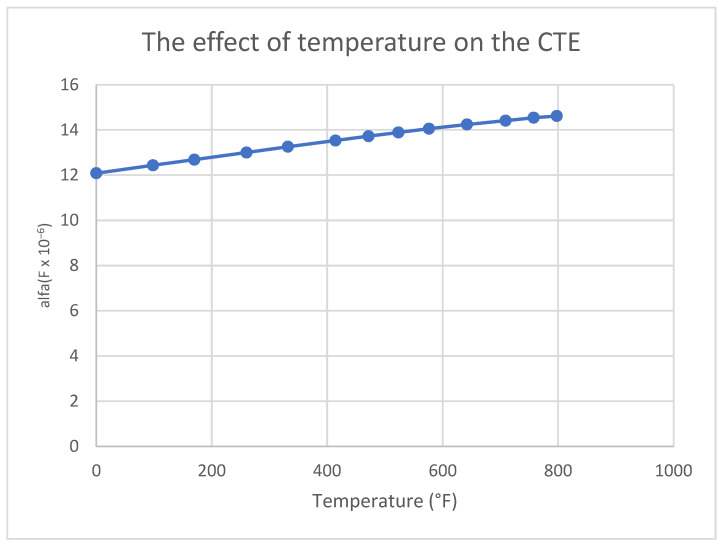
The effect of temperature on the coefficient of thermal expansion in aluminum 7075 [41].

**Figure 15:**
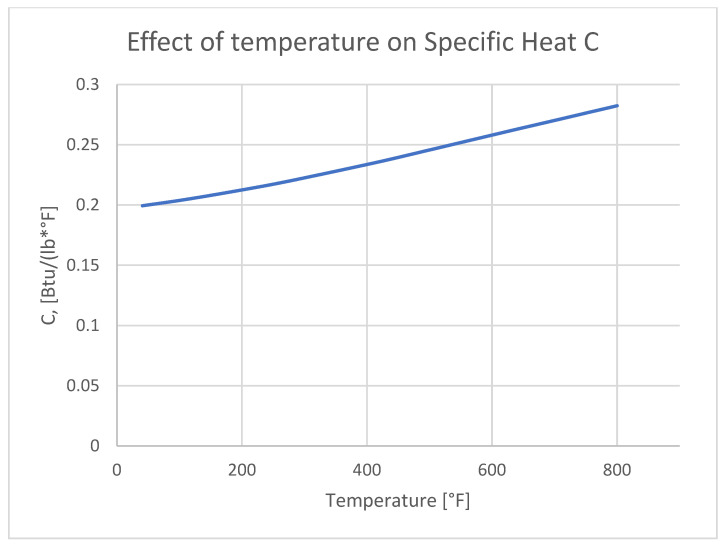
The effect of temperature on the Specific Heat (C) on aluminum 7075 [41].

The thermal expansion of aluminum (Figure 14) and its variation with temperature play a direct role in the formation of thermal stress, which subsequently lead to residual stress after bonding. The specific heat impacts the materials temperature change in time, introducing the temperature dependent graph allows a more accurate temperature distribution (Figure 15).

**Figure 16:**
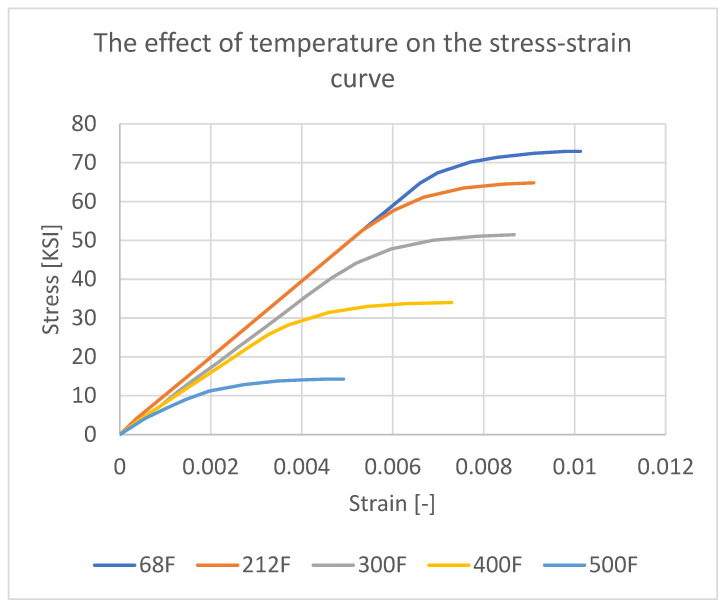
The effect of temperature on the stress–strain relation of the aluminum 7075 [41].

At elevated temperatures, aluminum enters the plastic range (Figure 16) under lower stress levels. The implemented material property cards account for the possibility of permanent deformation of aluminum.

**Figure 17:**
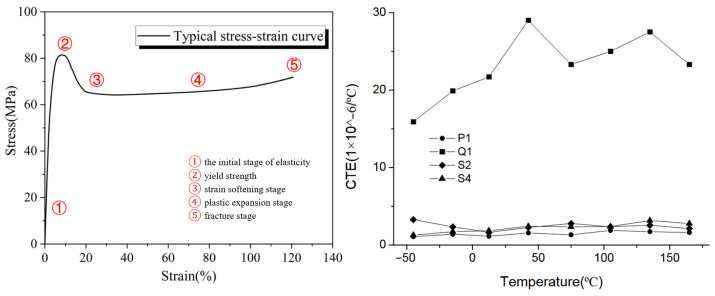
Stress–strain plot polycarbonate (PC) (**left**) [22]. The effect of temperature on the coefficient of thermal expansion of CFRP laminate (**right**) [43].

Consistent with ISO 11359-2 [42], CTE in polymers is defined for the solid state, and above Tg the material experiences complete stress relaxation and enters a softening/viscoelastic flow rather than a typical thermal expansion expressed by a coefficient. Vendors likewise specify CF/PC with a single room temperature CTE (e.g., 5 × 10^−6^ [°C^−1^] for TECATEC PC CW50—the laminate used here), so we model CF/PC with a constant effective CTE calibrated at room temperature for the welded laminate [42]. Figure 17 shows the correlation between temperature and carbon fibre-reinforced polymer matrix, which helps with understanding how the CTE reacts to temperature; the chaotic line, however, is hardly usable for simulation purposes. At first the material properties in the simulations were correlated with the datasheet provided by the distributor (Table 4); however, as indicated in Section 4.1., for these properties, especially, the most important one, the CTE did not correlate with the measured deflections. Hence, the implemented PC/CF properties were adjusted with the findings in [36,37].

**Figure 18:**
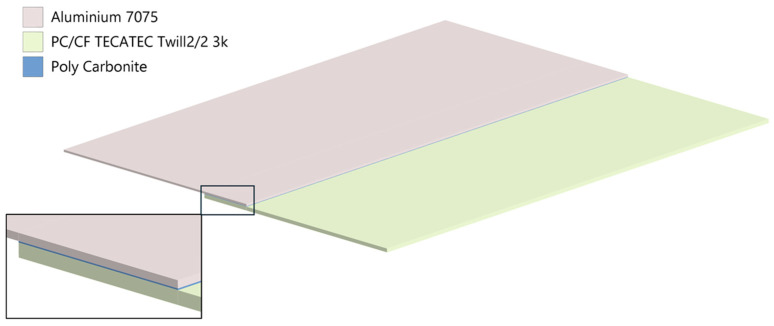
The CAD model.

**Figure 19:**
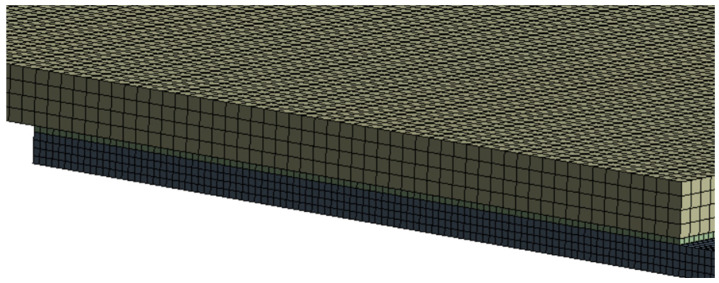
FEM mesh closeup.

The bonding material used is polycarbonate, an amorphous thermoplastic with excellent mechanical properties (Table 5), especially its impact resistance [27]. The key aspect of the study was leveraging the thermoplastic properties of polycarbonate by placing a 0.2 mm thick film (Figure 18 and Figure 19) between an aluminum plate and a PC/CF composite plate, followed by heating the joint area to the polycarbonate’s flow temperature of around 260 °C. As the polycarbonate film and the polycarbonate matrix in the laminate melt, they mix and penetrate the roughness of the mechanically prepared aluminum joint surface, forming a durable bond.

The challenge of this process is the difference in thermal expansion between the two materials. During heating to the flow temperature of polycarbonate, the materials expand and bond together. However, upon cooling to room temperature, the materials attempt to return to their original shapes, which is hindered by the weld, resulting in the formation of residual stress. This residual stress is the focus of this study.

The weld parameters, its behaviour under stress, and deformation due to temperature are influenced by the characteristics described below. These were defined in the Mechanical APDL environment.

**Figure 20:**
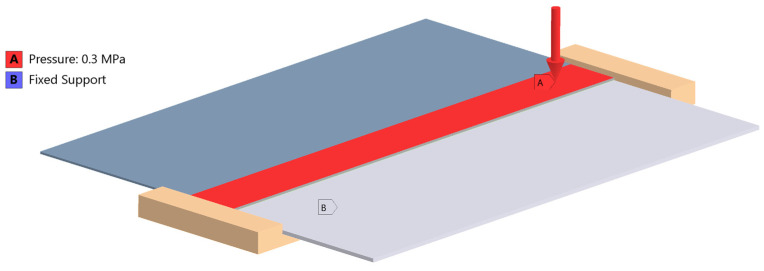
Pressure and temperature application in Ansys Mechanical 2023^®^.

An Ansys 2024R2 CAD model of the sample, followed by a FEM model (Figure 18, Figure 19 and Figure 20) were created. The boundary conditions present on the sample are the applied temperature, pressure and the frictional support (Figure 20).

Pressure and an elevated coefficient of friction (0.3) were additional factors used to stabilize the model [8,44].

### 5.1. Thermal Distribution

**Figure 21:**
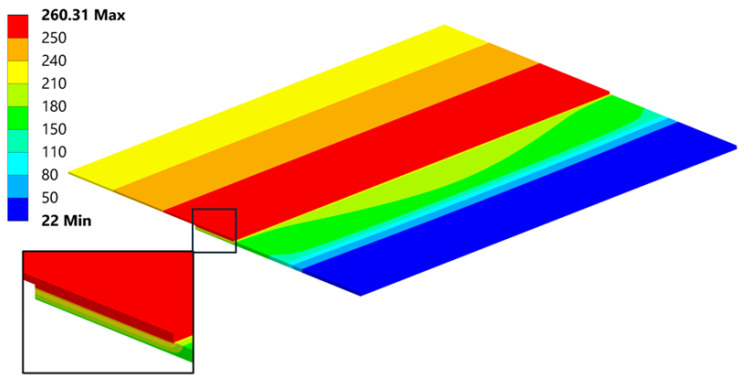
Temperature distribution at peak temperature [°C].

The first step in determining thermal stress and the resulting residual stress is a thermal analysis, which allows for identifying the temperature distribution within the heated components. Detailed material data implemented in the Ansys environment allowed for obtaining an accurate temperature distribution (Figure 21).

### 5.2. Deformation

**Figure 22:**
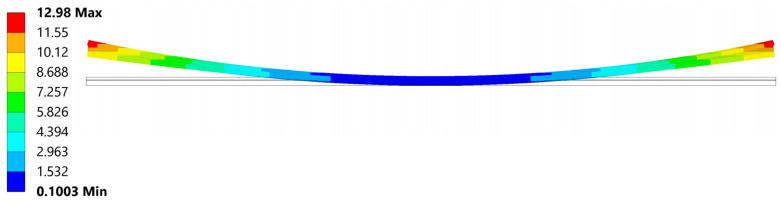
Deformation after cooling.

The most noticeable effect of residual stress present in welded parts after cooling is the resulting deformation (Figure 22). To reliably represent stress, efforts were made to achieve deformations as close as possible to those observed during the experiment. An analysis involving non-linear materials, non-linear contacts, and geometric non-linearity is overly sensitive to any changes.

The analysis produced a reliable deformation profile, with the magnitude of deformation being smaller than that observed in the experiment. This discrepancy is a result of the composite material card simplifications compared to reality, as well as uncertainties in the defined composite material’s response to temperature. Furthermore, even with the geometrical non-linearity allowed in the setting and large deformations permitted, the deformation results usually are observed to be smaller than those in real life.

### 5.3. Stress

This chapter will provide the stress results and discussion for the (20 °C–260 °C–20 °C) cycle only. Residual stress remaining after welding is crucial for the residual strength of the joint, which will be evaluated during static SLS tests. Understanding the residual stress within the joint, combined with the results of SLS tests, will provide insight into the total strength of the joint.

**Figure 23:**
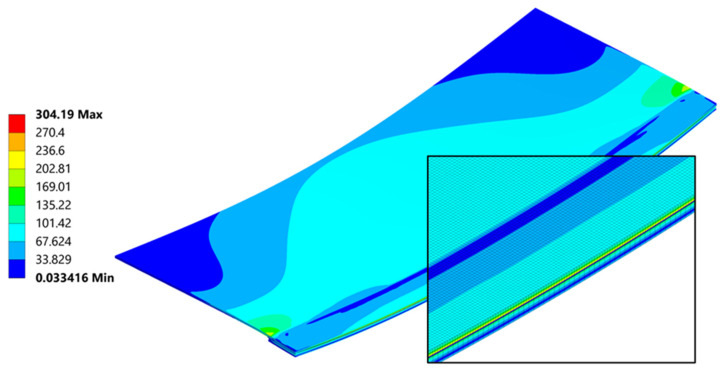
Cross section throughout the joint and a mesh including magnification of von Mises stress [MPa].

A general and overall stress contour of the von Mises results, seen in (Figure 23), presents information on the highest values and gives a rather simple description on what can be expected regarding the loading that the joint is carrying after welding. Far ends of the connection are bearing most of the forces involved quite similarly as seen in the empirical studies found in [45]. The von Mises stress results provide a general description and are used primarily for an introductory assessment. They do not provide a deep understanding of the direction or the Cauchy Stress Tensors, which are crucial for proper evaluation of the residual strength.

The cross section (Figure 23) provides insight into the differences in residual stress within the individual layers of the joint. Typically, during bending, a neutral line—where stress changes its direction—can be identified. In this case, due to the presence of three different materials bonded together at their maximum expansion temperature, and as they cool, they begin to compress. The carbon fibre plate reaches its room-temperature elongation (due to its lower thermal expansion coefficient), while the aluminum plate continues to shrink, transferring the compressive force into a bending moment. This composition creates particular ambiguity in the position of the neutral axis.

**Figure 24:**
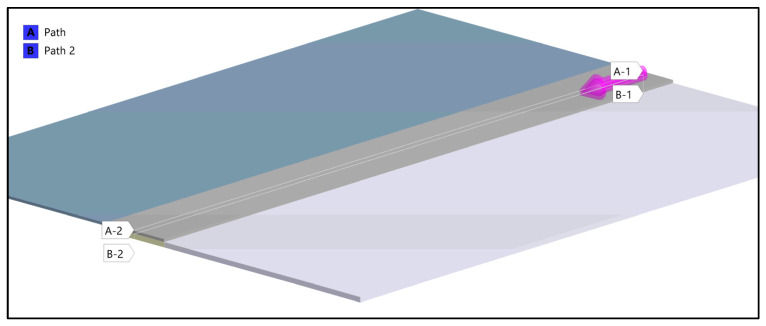
A stress path has been created on the inner (the bonding) and outer face of the aluminum plate.

**Figure 25:**
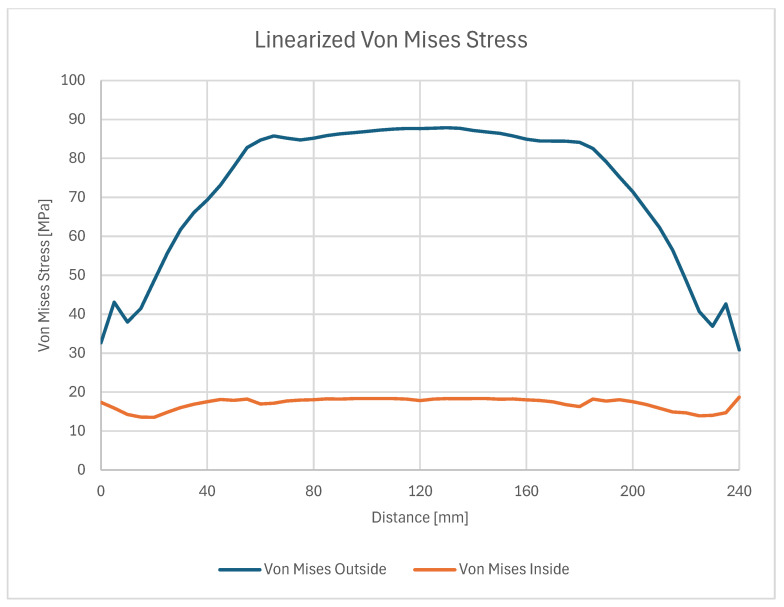
Linearized path stress (von Mises [MPa]).

On these paths, stress results are shown in (Figure 24) which gives a closer insight into the stress distribution in the joint itself and comparison with the stress on the outside plate, which correlates with [45]. The plotted stress path results are presented in (Figure 25).

Later, these samples will be subjected to various tests, such as uniaxial tension and the three-point bending test, which will provide the answers to the shear and normal residual strength of the weld. Because of that, the understanding of the residual stress components will be very useful.

**Figure 26:**
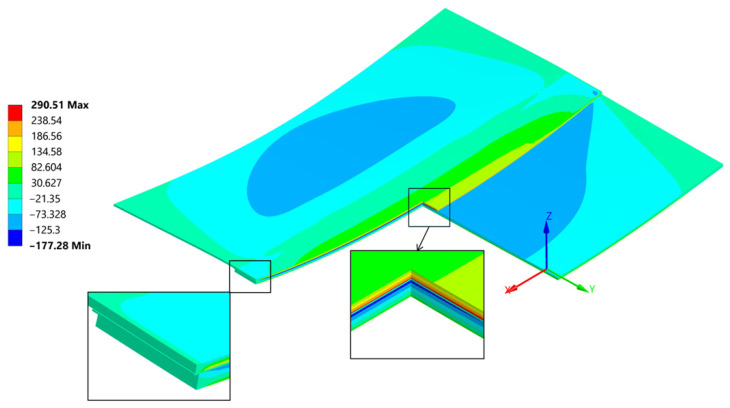
Normal Stress, σ_xx_ direction in the sample and the weld cut-out magnification.

Normal stress σ_xx_ reached a peak value of 290 MPa in tension and −177 MPa in compression (Figure 26). The compression occurs at the edge of the plate, where its highest values always appear when flexure is allowed [46].

**Figure 27:**
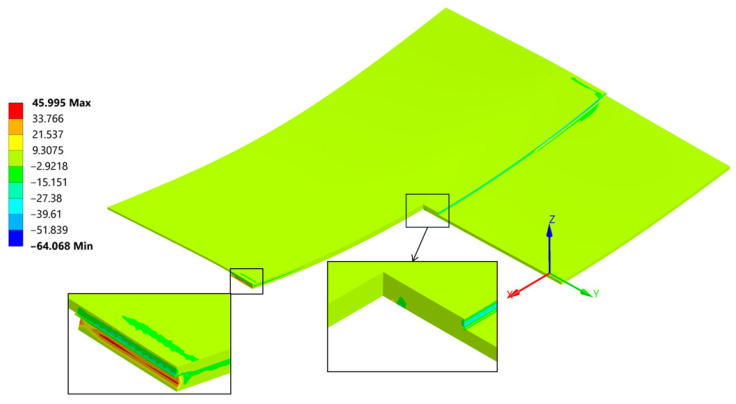
Normal stress σ_zz_ distribution inside the weld and at the weld edge.

The normal compressive stress σ_xx_ values calculated by hand at the edges in Section 4.2. averaged at around −191.8 MPa and were calculated considering the carbon plate Young’s modulus. As presented in (Figure 26), where only the bonded inside surfaces are highlighted. The normal peeling stress σ_zz_ derived only through FEM is presented in Figure 27.

These are considerably lower than the analytical assumptions. Such discrepancies arise because analytical calculations inherently use a highly simplified approach, determining the maximum possible extreme value, resulting in a very conservative estimate.

In contrast, FEM analyses provide results that are much closer to reality but remain as simulations that yield approximate values dependent on the adopted assumptions. In the case of composite analyses, it is generally estimated that the results are subject to an error margin of up to 20%.

**Figure 28:**
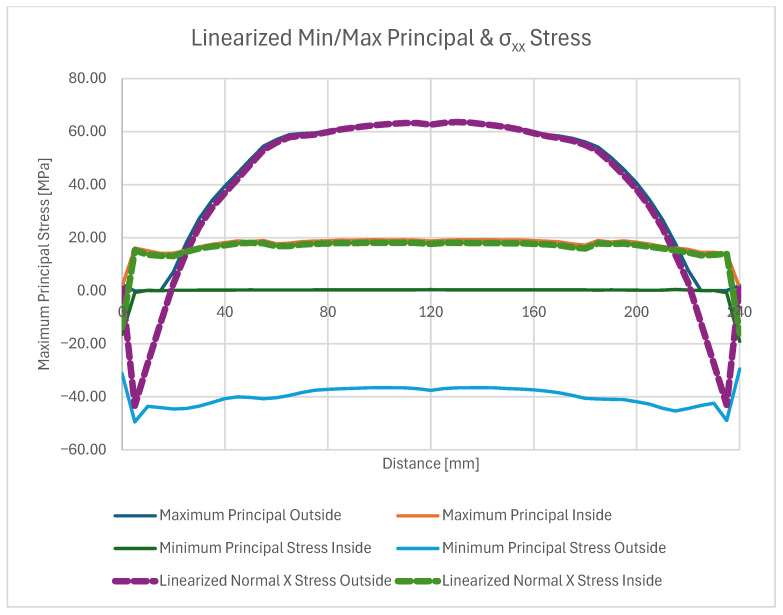
Linearized first and third principal stress.

**Figure 29:**
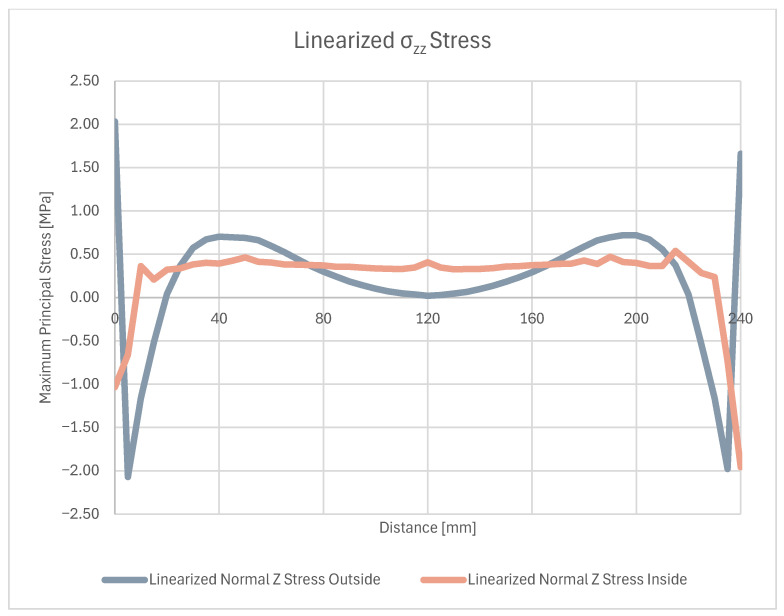
Linearized normal Z direction stress.

**Figure 30:**
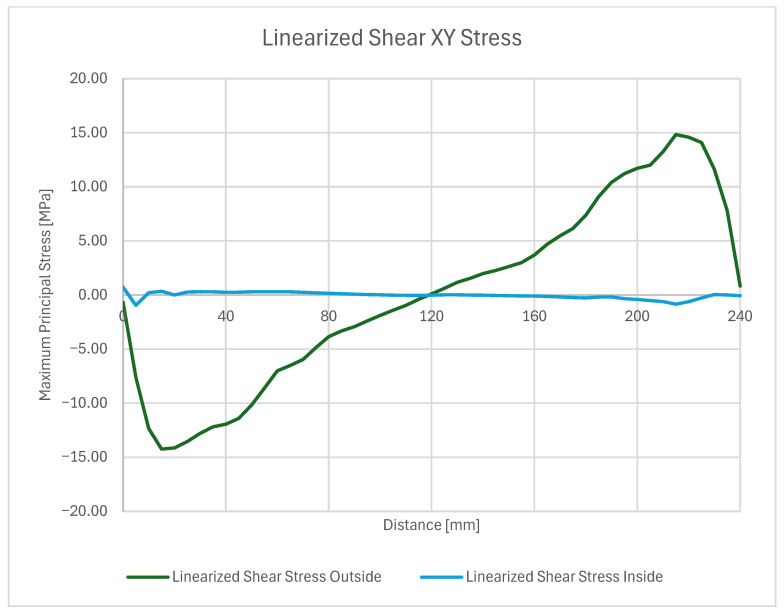
Linearized shear and normal stress plot.

**Figure 31:**
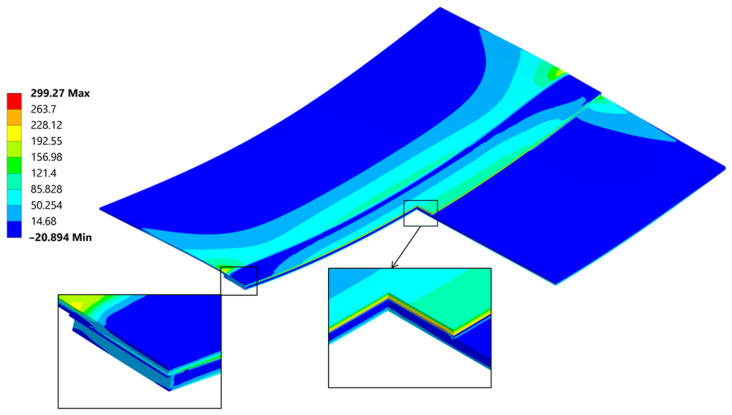
Maximum principal stress.

**Figure 32:**
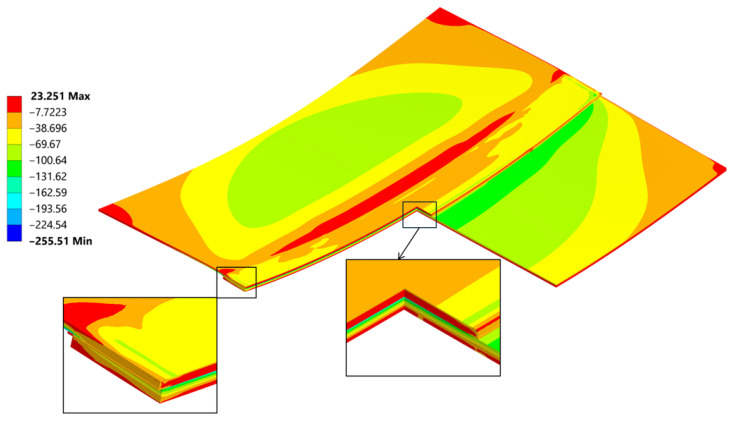
Minimum principal stress.

**Figure 33:**
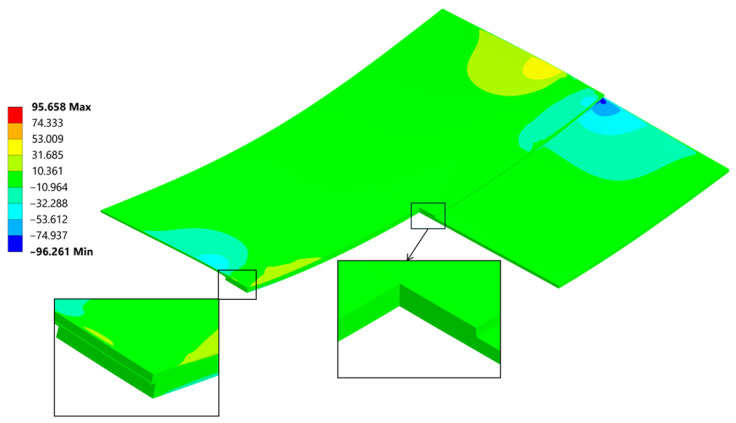
Shear stress result at the end of the 20 °C–260 °C–20 °C cycle.

Two stress paths were created and considered. One (A) is on the inner side of the bond, at the weld face, the other (B) is on the outer face of the composite. A thorough comparison of the stress along these paths is provided (Figure 28, Figure 29 and Figure 30) followed by full principal and shear stress plots (Figure 31, Figure 32 and Figure 33).

Similarly, in the case of shear stress, the obtained result deviates by approximately 8% from the analytical assumptions. The shear stress in the analytical calculation was estimated with the bending constrained. This simplification led to higher results.

**Figure 34:**
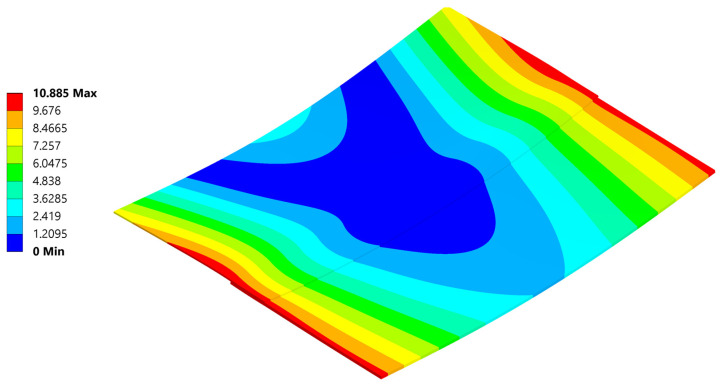
Plate deformation at the end of the 20 °C–220 °C–20 °C cycle.

**Figure 35:**
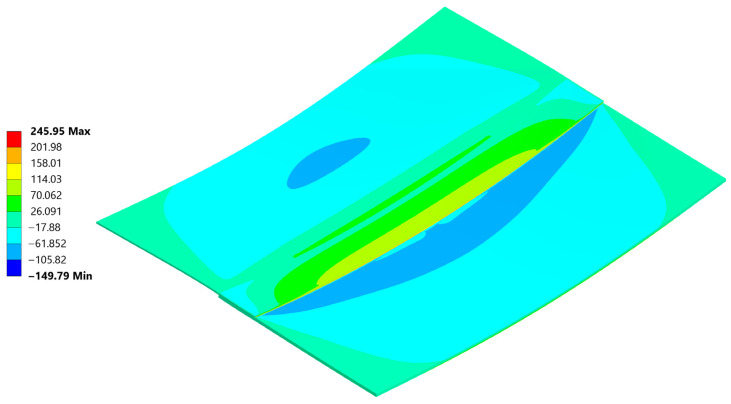
Normal σ_xx_ stress results at the end of the 20 °C–220 °C–20 °C cycle.

**Figure 36:**
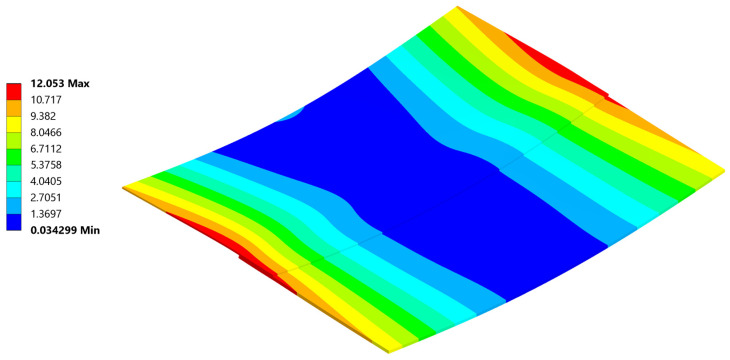
Deformation at the end of the 20 °C–240 °C–20 °C cycle.

**Figure 37:**
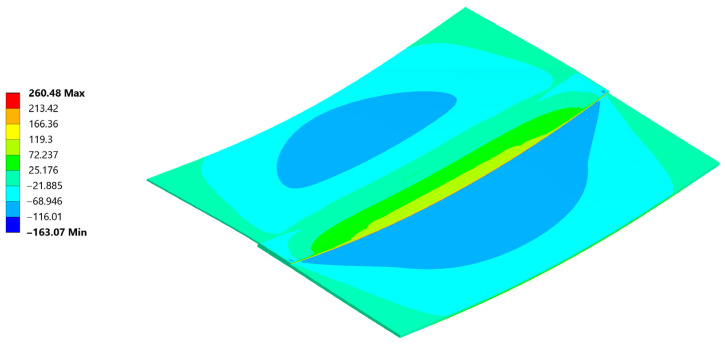
Normal σ_xx_ stress results at the end of the 20 °C–240 °C–20 °C cycle.

**Figure 38:**
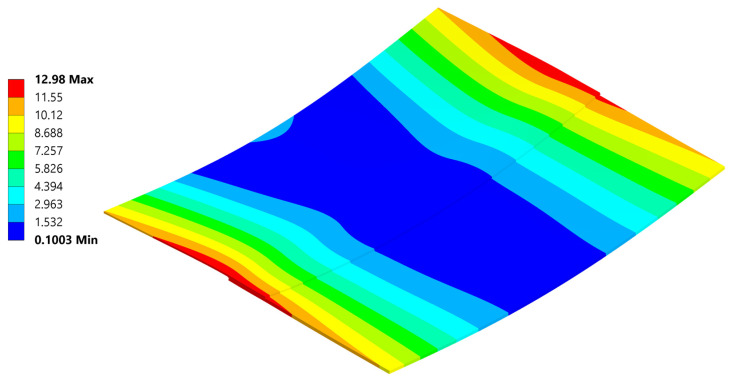
Deformation at the end of the 20 °C–260 °C–20 °C cycle.

**Figure 39:**
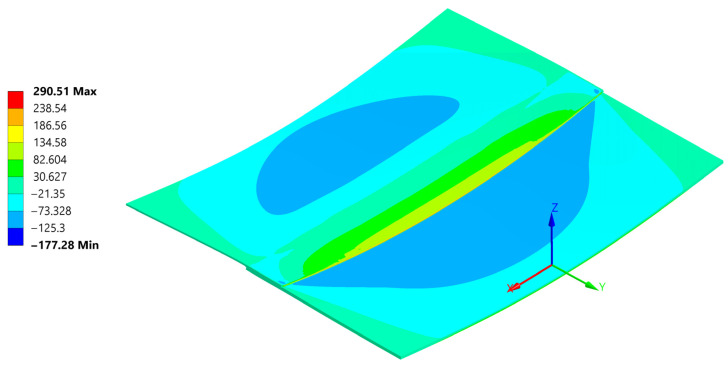
Normal σ_xx_ stress results at the end of the 20 °C–260 °C–20 °C cycle.

Figure 34, Figure 35, Figure 36, Figure 37, Figure 38 and Figure 39 showcase the stress and deformation differences for three cycle cases that were tested during welding.

Regarding the highest values of the shear stress at the edge, it must also be noted that the said peak abruptly drops when reaching the inwards of the weld, and when reaching the other edge, rises again, in a different direction; hence, the negative value. A similar phenomenon as with the normal stress is the “peeling” of the bond from the edge, and when the debonding begins, the stress value will translate with it.

The highest stress is present in the most far-reaching faces from the weld, which is somewhat in the middle. The plates that are being bended create a neutral axis [47] where stress is the lowest.

### 5.4. Deformation and σ_xx_ Stress Comparison Throughout Different Thermal Cycles

Below a direct comparison of deflection and σ_xx_ stress is presented for different thermal cycles. Results were obtained using Ansys 23R2 software.

## 6. Summary

The empirical data, hand calculations and finite element results are summarized in Table 6.

### 6.1. Conclusions

We developed and validated a computational workflow to quantify residual stresses in resistance-welded PC/CF–Al7075 single-lap joints, benchmarking a transient thermal to structural FEM against a Roark and Timoshenko bi-material analytical calculations and direct measurements.

The measured mid-span bow δ was 16.5–16.8 mm and remained constant throughout different temperature gradients (200–240 °C). The hand calculation resulted in a very similar mid-span bow δ at the ΔT = 240 °C with Mean Absolute Percentage Error (MAPE ≈ 11%), while the current FEM under predicted δ (MAPE = 30.25%) but captured a detailed stress distribution: edge-peaked peel σ_zz_ inside the bond increasing from −64 to 46 MPa with ΔT, and σ_xx_ in aluminum that show up to 12% more compressive stress than the analytical average.

Peel σ_zz_ and interface shear τ_xz_ peak at lap ends, consistent with Goland–Reissner (secondary bending edge peel) and Volkersen (end shear). These edge concentrations are the correct initiation sites for debonding in welded laps [48,49].

Residual stress implication on the durability is consistent with [18,50] where tensile residual stress increases the crack propagation rate and reduce the fatigue strength. In fracture terms, residual fields alter the effective driving force (energy release rate G): and accelerates, fatigue crack growth [51,52]. Because our joint shows edge-peaked σ_zz_/τ_xz_, the interface is pre-loaded in mixed Mode I/II at the free edges, elevating initiation risk under cycling—matching durability reports for lap joints [49].

Both analytical calculation and FEM show how bow δ is affected by ΔT, while the test does not. There are two reasons why this might be happening:

- PC flow/viscoelastic relaxation above Tg: Mismatch strains relax before lock-in, so the residual curvature is less temperature-sensitive, and the behaviour is not represented in the elastic hand calculation or current FEM.
- Al7075 hot softening/relaxation during dwell/early cooling reduces the recoverable bending moment, further flattening δ(ΔT) [53].

Analytical calculations report higher σ_xx_ stress than the FEM, this is because the prior is a section-averaged membrane stress; it cannot resolve edge concentrations. The 3-D FEM resolves secondary bending and shear-lag at the overlap ends; therefore, local σ_xx_ near the edges exceed the analytical averages—even when global δ is smaller [54,55].

For durability decisions, the edge peel and shear stress are governing. For the initiation analysis a mixed mode fracture mechanics such as VCCT must be performed to assess the fracture or, in this case, the debonding mode and calculating the release energy; these will be complementing static tests which will provide the necessary data.

### 6.2. Further Work

Future welds will be run with strain gauge instrumentation and with precise deformation measurements recorded with fibre optics welded in the overlap area. These will precisely measure the residual stress. Step-heating thermography will be complemented with higher-resolution ultrasonic inspection tests. The static tests, lap shear and three-point bending, will be executed to define the residual strength. Low-cycle fatigue tests will be performed with digital image correlation to identify initiation and debonding propagation trends, linking residual fields and edge stress concentrations to measured life.

## Figures and Tables

**Table 1:** Sample parameters.

Sample	T1 [°C]	T2 [°C]	t [mm] Thickness	Δ [mm] Displacement	L [mm] Length	B [mm] Width
AL-CPC-01	20	260	2.84	16.71	240	30
AL-CPC-02	20	240	3.02	16.97	240	30
AL-CPC-03	20	220	2.85	16.78	240	30

**Table 2:** Input data.

Parameter *	PC/CF (Layer a)	Al 7075 (Layer b)
Thickness t [mm]	1.8	1.0
E [GPa]	48	72
υ [-]	0.3	0.3
CTE α [1/K]	1.5 × 10^−6^ **	23 × 10^−6^

* The reported parameters in the table above are at room temperature. ** The PC/CF supplier states that the CTE = 5 × 10^−6^, this value however did not correlate with the results. Published CFRP measurements place the fibre direction CTE in the 1–2 × 10^−6^ [°C^−1^] range, and thermoplastic CFRP laminates (e.g., CF/PEEK) exhibit 3 × 10^−6^ [°C^−1^] near room temperature; our welded, fibre-rich CF/PC overlap, therefore, uses an effective in-plane CTE of 1.5 × 10^−6^ [°C^−1^], which is consistent with the fibre dominated literature and results in a similar deflection value as the measured samples [36,37].

**Table 3:** Shear stress in the joint.

ΔT [K]	Curvature κ [1/m]	Bow δ Calculated [mm]	Bow δ Measured [mm]	Error [%]	$\sigma_{xx}$ [MPa]
200	−1.872	13.48	16.779	−19.66	−159.9
220	−2.059	14.83	16.542	−10.35	−175.8
240	−2.246	16.17	16.71	−3.23	−191.8

**Table 4:** CFPC datasheet (ensingerplastics.com; accessed on 19 September 2024).

Parameter	Value	Unit
Fibre type	carbon HT 3 k	-
Fibre architecture	twill 2/2	-
Fibre areal weight	200	g/m^2^
Tensile strength	440	MPa
Modulus of elasticity (tensile test)	48,000	MPa
Flexural strength	615	MPa
Modulus of elasticity (flexural test)	48,000	MPa
Compression strength	215	MPa
Glass transition temperature	143	°C
Service temperature (short term)	140	°C
Service temperature (long term)	120	°C
Thermal expansion (CLTE)	5	10^−6^ K^−1^
Drying temperature	120	°C
Drying time	3	h

**Table 5:** Polycarbonate parameters.

Property	Value
Young Modulus [GPa]	2.5
Poisson’s Coefficient	0.34
Coefficient of thermal expansion	0.65 × 10^−4^
Heat Conductivity [m^−1^K^−1^]	0.2
Specific Heat [kJ kg^−1^K^−1]^	1.2

**Table 6:** Summary.

Parameter	ΔT = 200 °C	ΔT = 220 °C	ΔT = 240 °C
δ test [mm]	16.78	16.54	16.71
δ analytical [mm]	13.48	14.83	16.17
δ error analytical [%]	−19.67	−10.34	−3.23
δ FEM [mm]	10.88	12.05	12.98
δ error FEM [%]	−34.88	−30.5	−21.21
σ_xx_ analytical [MPa]	−159.9	−175.8	−191.8
Linearized σ_xx_ FEM [MPa]	43.21	55.6	63.63
Δσ_xx_ FEM vs. analytical [%]	1.08	7.36	10.28
Linearized σ_zz_ FEM (edge) [MPa]	−1.75	−1.93	−2.07

## Data Availability

The original contributions presented in this study are included in the article. Further inquiries can be directed to the corresponding author.
